# Measuring the preparedness of health facilities to deliver emergency obstetric care in a South African district

**DOI:** 10.1371/journal.pone.0194576

**Published:** 2018-03-29

**Authors:** Siphiwe Bridget Pearl Thwala, Duane Blaauw, Freddie Ssengooba

**Affiliations:** 1 Centre for Health Policy, School of Public Health, Faculty of Health Sciences, University of the Witwatersrand; Johannesburg, South Africa; 2 Faculty of Health Sciences, University of Swaziland; Mbabane, Swaziland; 3 School of Public Health, Makerere University, Kampala, Uganda; National Institute of Health, ITALY

## Abstract

**Background:**

Improving the delivery of emergency obstetric care (EmNOC) remains critical in addressing direct causes of maternal mortality. United Nations (UN) agencies have promoted standard methods for evaluating the availability of EmNOC facilities although modifications have been proposed by others. This study presents an assessment of the preparedness of public health facilities to provide EmNOC using these methods in one South African district with a persistently high maternal mortality ratio.

**Methods:**

Data collection took place in the final quarter of 2014. Cross-sectional surveys were conducted to classify the 7 hospitals and 8 community health centres (CHCs) in the district as either basic EmNOC (BEmNOC) or comprehensive EmNOC (CEmNOC) facilities using UN EmNOC signal functions. The required density of EmNOC facilities was calculated using UN norms. We also assessed the availability of EmNOC personnel, resuscitation equipment, drugs, fluids, and protocols at each facility. The workload of skilled EmNOC providers at hospitals and CHCs was compared.

**Results:**

All 7 hospitals in the district were classified as CEmNOC facilities, but none of the 8 CHCs performed all required signal functions to be classified as BEmNOC facilities. UN norms indicated that 25 EmNOC facilities were required for the district population, 5 of which should be CEmNOCs. None of the facilities had 100% of items on the EmNOC checklists. Hospital midwives delivered an average of 36.4±14.3 deliveries each per month compared to only 7.9±3.2 for CHC midwives (p<0.001).

**Conclusions:**

The analysis indicated a shortfall of EmNOC facilities in the district. Full EmNOC services were centralised to hospitals to assure patient safety even though national policy guidelines sanction more decentralisation to CHCs. Studies measuring EmNOC availability need to consider facility opening hours, capacity and staffing in addition to the demonstrated performance of signal functions.

## Introduction

Significant progress has been made to combat maternal mortality. From 1990 to 2015, the maternal mortality ratio (MMR) reduced by 45% globally, and by 49% in sub-Saharan Africa [1]. While this improvement is commendable, evaluation of the millennium development goals (MDGs) achievement by region has shown that the MMR remains very high in sub-Saharan Africa [2]. For the new sustainable development goals (SDGs), the world has resolved to end all preventable maternal mortality, and committed to the ambitious goal of reducing the global MMR to 70 per 100 000 live births or less, while making sure that no country has an MMR above 140, by the year 2030 [3]. Realisation of these targets requires immediate and concerted efforts [4].

Emergency obstetric and neonatal care (EmNOC) services are effective in dealing with direct causes of maternal mortality and therefore necessary to achieve these global goals [5–8]. Measurement of the availability of EmNOC identifies the number of health facilities that are able to provide basic or comprehensive EmNOC services, and is one of eight key process indicators (Table 1) developed by the World Health Organization (WHO) and other United Nations (UN) agencies to monitor EmNOC services provision [9]. Measurement of this indicator requires that facilities demonstrate recent performance of seven so-called EmNOC ‘signal functions’ to be recognised as a basic EmNOC (BEmNOC) facility, or nine specified ‘signal functions’ to be considered a comprehensive EmNOC (CEmNOC) facility. The UN handbook originally proposed that four BEmNOC and one EmNOC facilities are required per 500 000 population [9]. This standardised approach to measuring the availability of EmNOC uses basic, easily collected information, and has been applied in many countries, making it possible to compare them [10–13].

**Table 1:** UN indicators for monitoring EmNOC [9].

Indicator 1	Availability of EmNOC
Indicator 2	Geographical distribution of EmNOC facilities
Indicator 3	Proportion of all births in EmNOC facilities
Indicator 4	Met need for EmNOC
Indicator 5	Caesarean sections as a proportion of all births
Indicator 6	Direct obstetric case fatality rate
Indicator 7	Intrapartum and very early neonatal death rate
Indicator 8	Proportion of deaths due to indirect causes in EmNOC facilities

However, a number of scholars and practitioners have argued that the UN EmNOC approach to measuring the availability of EmNOC facilities, the first EmNOC process indicator, requires modification and expansion [14–16]. The UN EmNOC Handbook does not discriminate between the size and therefore demand on facilities and this is a gap as smaller facilities (e.g. health centres) are counted the same as larger facilities (e.g. referral hospitals). Measuring skilled provider distribution across facilities and geographical access along EmNOC availability could potentially redress this gap [15]. Another criticism of the ‘signal functions’ is that they do not consider that identified EmNOC facilities may not be open 24 hours, always have skilled staff on duty, or have sufficient capacity to deal with EmNOC cases [15, 17, 18]. Gabrysch et al (2012) proposed additional signal functions, such as the availability of fluids to control hypovolemic shock, inorder to improve the measurement of EmNOC service availability [16]. Consensus on these modifications is emerging as several studies have measured the availability of EmNOC in facilities using both the UN EmNOC signal functions and these additional signal functions reflecting other aspects of availability [19–21]. Furthermore, the UN EmNOC handbook changed the density of facilities indicator norm to at least 5 EmNOC facilities per 500 000 population from facilities per births previously used in the world health report of 2005 [9, 22]. Others argued that the norm should be calculated per 20 000 live births because different populations have different fertility and different corresponding needs [10, 15].

In South Africa, an unacceptably high MMR persists at 133 per 100 000 live births[23]. Therefore, the country has not achieve the MDG goal of reducing maternal deaths by 75% between 1990 and 2015 [1, 24–27]. Some progress has been made recently in decreasing HIV-related maternal mortality, but more needs to be done to improve EmNOC services and address the direct causes of maternal mortality to achieve any further meaningful reduction of the MMR [24, 27]. Surprisingly, there have been very few studies using the UN process indicators to evaluate EmNOC services in South Africa[28]. A recent paper evaluated the availability of EmNOC services in 12 districts in South Africa and found that important aspects of both basic and comprehensive EmNOC are not readily available in many public health facilities [21].

Given the persistently high MMR of 113 per 100 000 live births in the Gauteng Province of South Africa, the provincial government requested the assessment of the availability EmNOC in one priority district with a MMR of 169 per 100 000 live births, significantly higher than the provincial average [26]. This study aimed to measure the availability of EmNOC services in the district using the UN EmNOC signal function method adapted to suit the district EmNOC needs. We also sought to critically appraise the UN method of measurement of EmNOC availability in the district.

## Materials and methods

All 15 designated public health facilities in the district were included in the study: 8 community health centres (CHCs), 4 regional hospitals, 2 district hospitals, and 1 tertiary hospital. The district also has 87 smaller clinics and 42 ward based primary health care outreach teams offering mobile services to 30 wards which were not included in this evaluation [29]. The department of health (DoH) does not promote deliveries at these lower clinics or consider them as EmNOC facilities. They do not have labour wards or midwife obstetric units (MOUs) and mainly provide outpatient services. All private health facilities were also excluded. The district had a total population of 3 284 630 in 2014, of which 2 446 204 (about 74%) were without private health insurance and assumed to make use of the public sector facilities [26, 30].

Data collection took place in the last quarter of 2014. Health facilities were surveyed for performance of the UN BEmNOC and CEmNOC signal functions (Table 2) in the 3 months preceding data collection, using tools adapted from the Averting Maternal Disability and Death (AMDD) manual [9, 31]. Maternity registers were viewed for evidence of the signal functions performed. Where signal functions were not identified from the registers, labour ward nurse managers were asked to confirm that these signal functions had not been performed, and asked why they had not been performed. Dates of EmNOC drills in the previous year were recorded to establish the frequency of performance of resuscitation drills to safeguard quality of care. The number of births for 12 months were also recorded from all facilities.

**Table 2:** UN EmNOC signal functions.

EmNOC level	Signal functions	
**Basic** **EmNOC**	1. Administer parenteral antibiotics 2. Administer uterotonic drugs (e.g. parenteral oxytocin) 3. Administer parenteral anticonvulsants for pre-eclampsia and eclampsia (e.g. Mg_2_SO_4_) 4. Manually remove the placenta 5. Remove retained products of conception (e.g. manual vacuum extraction, dilatation and curettage 6. Perform assisted delivery (e.g. vacuum extraction and forceps delivery 7. Perform basic neonatal resuscitation (e.g. with bag and mask)	**Total score = 7**
**Comprehensive** **EmNOC**	8. Perform surgery 9. Perform blood transfusion	**Total score = 9**

Facilities were classified as BEmNOC and CEmNOC facilities if they had provided all 7 BeMNOC or 9 CEmNOC signal functions respectively (Table 1). The required number of EmNOC facilities in the district was calculated against the UN benchmarks of 5 EmNOC required, of which at least 1 ought to be a CEmNOC facility of per 500 000 population as follows [9];

$$Overall\;required\;EmNOC\;facilities=\frac{Public\;sector\;population}{500\;000}x5$$

The required density of EmNOC was also calculated per 20 000 births for comparison;

$$Overall\;required\;EmNOC\;facilities=\frac{Number\;of\;public\;sector\;births}{20\;000}x\;5$$

Other indicators to measure EmNOC included are based on the current literature on measuring EmNOC availability, e.g. availability of skilled staff, opening hours of facilities, and the presence of electricity in facilities [16].

To benchmark skilled staff availability, a critical mass of 5 advanced midwives and 5 midwives in CHCs at any given time to achieve safe dispensation of maternity care including BEmNOC services as proposed by a south African maternal health expert was used [32]. This benchmark was used in the absence of staffing norms developed by the South African government that could have been used to benchmark staffing levels for the safe delivery of basic EmNOC in CHCs.

Multiple checklists were used to measure the availability of essential elements of EmNOC care (Table 3). For EmNOC human resources, health professionals that had formal training in maternity care (obstetrics and midwifery) were regarded as skilled providers. Only skilled providers working in maternity were counted. The number, category, and level of training of providers were obtained from the heads of obstetric departments and maternity nursing managers. Availability of selected EmNOC drugs and fluids was assessed on the day of data collection. A score of 1 was given for each drug or fluid if physically present, and 0 if not physically present in the labour ward, or present but expired. The physical presence of useable whole blood was also assessed in all hospitals. Availability of adult and neonate resuscitation equipment was audited by determining if items were present and functional in the respective resuscitation trolleys. Resuscitation equipment items were scored 1 if available and functional, and 0 otherwise. We also evaluated if 10 selected EmNOC protocols were present and readily accessible for use in the labour ward, displayed on the wall for example, on the day of data collection. The protocols were scored 1 if readily available and 0 otherwise. We also enquired from the labour ward manager about the frequency and dates of both adult and neonatal resuscitation drills in 2013 to assess maintenance of the quality of EmNOC skills by providers.

**Table 3:** Summary of tools and indicators.

General measures	Blood, drugs, fluids and equipment availability measures				Quality control measures	
	Drugs	Blood and Fluids	Adult resuscitation equipment	Neonate resuscitation equipment	Availability of protocols	Performance of resuscitation drills
1. Facility opening hours 2. Availability of electricity 3. Tap water for washing	1. Oxytocin 2. Syntomentrine 3. Ergometrine 4. Magnesium sulphate 5. Rivotril / diazepam / equivalent 6. Misoprostol 7. Insulin 8. 50% Glucose 9. Parenteral antibiotics	**Blood** 1. Whole blood **Fluids** 1. Normal saline 2. Ringers lactate 3. 5% Dextrose	1. Suction machine 2. Adult suction catheter 3. Laryngoscope set 4. Endo-tracheal tube 5. Oxygen 6. Magill’s forceps 7. Airway 8. Adult ambubag 9. Intravenous cannula 10. Fluid administration set 11. Blood administration set 12. Stethoscope 13. Sphygmomanometer 14. Glucostix 15. Glucometer	1. Suction machine 2. Neonate suction catheters 3. Laryngoscope set 4. Endo-tracheal tube 5. Oxygen 6. Incubator 7. Radiant infant warmer 8. Neonatal ambubag 9. Airway 10. Stethescope	1. Eclampsia 2. Postpartum haemorrhage 3. Active management of the 3^rd^ stage of labour 4. Shoulder dystocia 5. Cord prolapse 6. Obstructed labour 7. Puerperal sepsis 8. Antepartum haemorrhage 9. Retained placenta 10. Acute collapse/Adult CPR/ unconscious patient	1. Adult resuscitation drills 2. Neonate resuscitation drills 3. Maternal morbidity and mortality meetings
**3 Items**	**9 Items, Score = 9**	**3 fluid Items, Score = 3**	**15 Items, Score = 15**	**10 Items, Score = 10**	**10 Items, Score = 10**	**3 Items**

Data from the checklists were entered and analysed using Stata v13 (StataCorp). Scores were converted to percentages of the total maximum checklist score for easier comparison. Means and standard deviations were calculated for CHCs and hospitals in the district respectively. Score means from the various checklists were compared using Krusskal-Wallis and Mann-Whitney tests.

Ethical clearance to conduct the study was obtained from the Human Research Ethics Committee of the University of the Witwatersrand. Approval to conduct the study was also obtained from the district research committee of the department of health, and each health facility gave permission for data collection. Labour ward nurse managers signed informed consent before giving any information to researchers. All participants were assured of their right to withdraw participation at any stage without prejudice.

## Results

### Classification of EmNOC facilities

Based on their performance of the UN signal functions, only 7 (46.7%) of the facilities in the district would be classified as EmNOC facilities. All 7 of the hospitals in the district fulfilled the requirements to be classified as CEmNOC facilities, and the same 7 provided BEmNOC services. The 8 CHCs performed some of the BEmNOC signal functions, but none of them performed all 7 to earn classification as a UN EmNOC facility (Table 4). All CHCs offered parenteral anti-convulsants, uterotonic drugs, and performed neonatal resuscitation. Five CHCs (62.5%) had given parenteral antibiotics, while only two (25.0%) performed manual removal of the placenta or remove retained products of conception in the previous 3 months. None of the CHCs performed assisted vaginal deliveries (Table 4). The primary reason given by all CHCs for not performing some of the basic signal functions was district policy which stated that CHCs should not perform assisted deliveries, manual removal of placenta, or removal of retained products because of inadequate resources in the CHCs to safely support these activities. This was despite national policy guidelines that sanctioned the performance of these signal functions at the CHC level [33]. Nurses in facilities (CHCs) that did not administer parenteral antibiotics recorded that it was out of their scope of practice to prescribe them to patients even when indicated. They therefore referred such patients to higher levels of care. Other less common reasons provided were: lack of equipment and supplies; training deficiencies among staff; management problems; as well as no indication/ requirement for the signal function from service users.

**Table 4:** Percentage of facilities performing signal functions and availability of general measures.

Category	Indicator	% of CHCs (n = 8)	% of Hospitals (n = 7)	% of Total (n = 15)
BEmNOC signal functions	Administer uterotonic drugs	100.0%	100.0%	100.0%
	Administer parenteral anticonvulsants	100.0%	100.0%	100.0%
	Perform basic neonatal resuscitation	100.0%	100.0%	100.0%
	Administer parenteral antibiotics	62.5%	100.0%	80.0%
	Manual removal of placenta	25.0%	100.0%	60.0%
	Remove retained products	25.0%	100.0%	60.0%
	Perform assisted delivery	0.0%	100.0%	46.7%
CEmNOC signal functions	Perform caesarean section	0.0%	100.0%	100.0%
	Perform blood transfusion	0.0%	100.0%	100.0%
General measures	Open 24 hours, 7 days a week	100.0%	100.0%	100.0%
	Electricity	100.0%	100.0%	100.0%
	Tap water for washing	87.5%	100.0%	93.3%

All 15 health facilities in the district were open 24 hours a day, 7 days a week. Electricity was also available in all facilities. Only one CHC did not have running water (Table 4).

### Density of EmNOC facilities

According to the UN EmNOC guidelines there should be at least 5 EmNOC facilities per 500 000 people of which a minimum of 1 must be a CEmNOC facility [9]. A total of about 25 EmNOC facilities are required for the public sector population in the district, indicating a shortfall of at least 18 EmNOC facilities (Table 5). If we use the UN EmNOC Handbook configuration of 4 BEmNOC facilities and 1 CEmNOC facility per 500 000 people, 25 EmNOC facilities in total are required of which at least 5 are supposed to provide CEmNOC and 20 provide BEmNOC. Table 5 compares the current number of EmNOC facilities to the number that should be available using this norm. There was therefore an adequate number of CEmNOC facilities in the district (7), but a significant undersupply of BEmNOC facilities. Even if all 8 of the available CHCs were upgraded to function as BEmNOC facilities, there would still be a shortfall of at least 10 BEmNOC facilities in the district for the current population according to this analysis.

**Table 5:** Required density of EmNOC facilities.

Category	Current facilities	Analysis per 500 000 population^‡^		Analysis per 20 000 births^‡^	
		Target	Surplus/deficit	Target	Surplus/deficit
**BEmNOC**	7	19.6	-12.6	12.9	-5.9
**CEmNOC**	7	4.9	+2.1	3.2	+3.8
**Total**	7	24.5	-17.5	16.1	-9.1

‡ For 2 446 204 uninsured population in the district with 64 544 births [26]

There were 64544 births recorded from all 15 facilities in the district. When the norms per 20 000 births rather than 500 000 population are used, the shortfall in EmNOC facilities is less marked at 9 facilities Table 5. There is a deficit of at least 6 BEmNOC facilities.

### Availability of skilled EmNOC providers

Skilled EmNOC providers included general professional nurses whose formal training included midwifery, specialised professional nurses with advanced midwifery training, and doctors. However, only advanced midwives and doctors have the requisite formal training to perform all 7 UN BEmNOC signal functions.

Advanced midwives were available in all 15 facilities, but not 24 hours per day or 7 times per week. Only 12.5% of CHCs and 42.9% of hospitals were able to provide such cover Table 6. According to duty rosters, the number of advanced midwives in CHCs ranged from 1 to 4 during the day, and from 0 to 2 at night. The corresponding figures for hospitals were 1 to 3 during the day, and 0 to 1 at night. Absence of an advanced midwife on duty meant that the facility had an impaired ability to perform all BEmNOC signal functions, unless a doctor was present. More midwives and advanced midwifes were employed in CHCs yet there were markedly more deliveries in hospitals. This made the workload of hospital midwives significantly higher than those at CHCs (Table 6). CHCs had a doctor available some days of the week, mostly during the daytime. All hospitals had doctors available 24 hours for all days of the week, and only two hospitals did not also have a specialist working in maternity. The presence of doctors was particularly thin afterhours on weekdays and on weekends, risking potentially compromise of the performance of EmNOC signal functions to all women needing the service in hospitals. Hospital midwives delivered an average of 36.4±14.3 deliveries each per month compared to only 7.9±3.2 for CHC midwives (p<0.001, Mann-Whitney).

**Table 6:** EmNOC human resources and workload.

Category	Indicator	CHCs (n = 8)	Hospitals (n = 7)	Total (N = 15)
**Midwives**	Total number of midwives	140	128	268
	% Facilities with midwife available 24/7 on site	100.0%	100.0%	100.0%
**Advanced** **Midwives**	Total number of advanced midwives	56	34	90
	Number of advanced midwives per day shift (range)	1–4	1–3	1–4
	Number of advanced midwives per night shift (range)	0–2	0–1	0–2
	% Facilities with advanced midwife available 24/7 on site	12.5%	42.9%	26.7%
**Doctors**	Total number of doctors working fulltime	0	89	89
	Number of doctors per day shift (range)	0–3	5–18	0–18
	Number of doctors per night shift/ weekend	0	1–3	0–3
	Total number of obstetric consultants	0	9	9
	% Facilities with obstetric consultant	0.0%	71.4%	33.3%
	% Facilities with doctor available 24/7 on site	0.0%	100.0%	46.7%
	% Facilities with obstetrician available 24/7 on site	0.0%	0.0%	0.0%
**Workload**	Number of deliveries in month of data collection	1090	4786	5876
	Deliveries per midwife (mean ± sd)	7.9 ± 3.2	36.4 ± 14.3	21.2 ± 17.6

Since South Africa has not yet developed staffing norms for the safe delivery of basic EmNOC in CHCs, although it has been suggested that a minimal staff of 5 advanced midwives and 5 midwives are required in CHCs at any given time to dispense safe maternity care [34]. None of the CHCs in the district achieved this critical mass.

### Availability of EmNOC drugs and equipment

**Fig 1:**
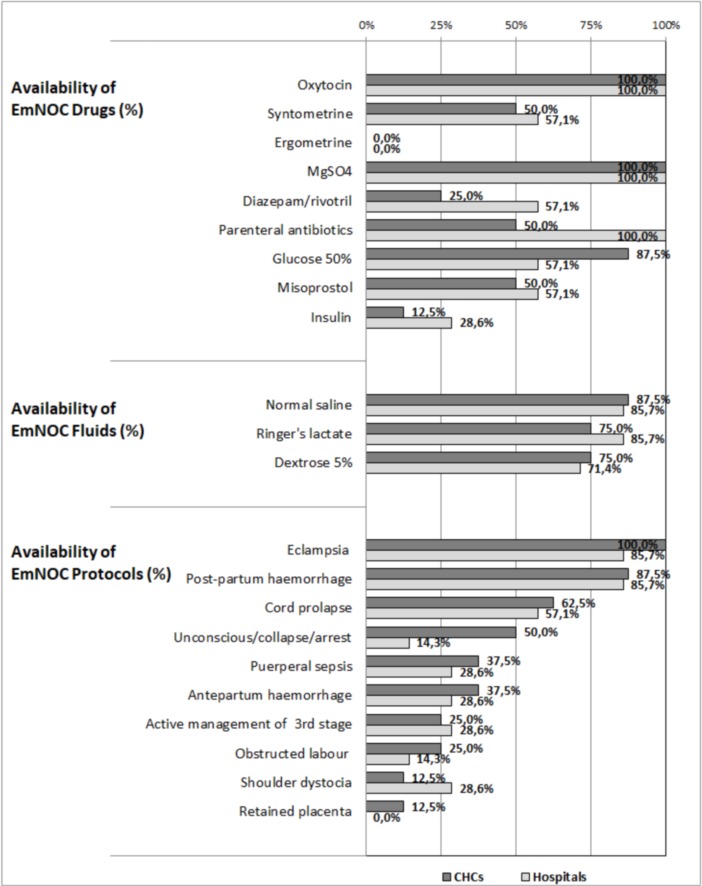
Availability of individual drugs, fluids, and protocols across facilities.

All hospitals had whole blood available for transfusion on the day of data collection (Table 7). We did not, however, ascertain the availability of different blood groups. None of the facilities in the district scored 100.0% for availability of either EmNOC drugs or fluids. The average drug availability score in the district was 56.7% ± 13.3%, ranging from a minimum of 33.3% to a maximum of 88.9%, with higher scores in hospitals than CHCs. The mean score for fluids was 80.0% ± 27.7% with a range of 33.3% to 100.0%. Fig 1 shows the details of the drugs and fluids available in CHCs and hospitals. Parenteral antibiotics, syntometrine, diazepam and insulin were not available in many CHCs, but the figures for hospitals were also surprisingly low. Ergometrine was not available in any of the facilities.

**Table 7:** EmNOC equipment and supplies.

Category	CHCs (n = 8)	Hospitals (n = 7)	Total (n = 15)
% Facilities with whole blood	0.0%	100.0%	100.0%
Drug score, as % out of 9 (mean ± sd)	59.4 ± 14.4	65.6 ± 12.2	56.7 ± 13.3
Fluid score, as % out of 3(mean ± sd)	80.0 ± 27.7	81.7 ± 30.7	80.0 ±27.7
Adult resuscitation equipment score, as % out of 15 (mean ± sd)	78.7±9.3	79.3± 14.7	78.7±11.3
Neonate resuscitation equipment score, as % out of 13 (mean ± sd)	90.0±11.0)	79.0±13.0	85.0±13.0

The mean score for the availability of adult resuscitation equipment was 78.7% ± 11.3% (Table 7). Individual facility scores ranged from 60.0% - 100.0%. The average score for neonatal resuscitation equipment was 85.0% ± 13.0% with a range of 60.0% - 100.0%. Surprisingly, the least available adult resuscitation tool was a functional stethoscope as only 5 facilities (33.3%) had one readily available for use in the resuscitation trolley on data collection day. Doctors generally had their own stethoscopes with them at all times but midwives and nurses did not, which was problematic when confronted with an emergency. When probed nurse-managers reported that stethoscopes often got lost. They were therefore no longer kept in the trolleys, but locked up in the nursing sister’s office, although this was contrary to standard operating procedures.

### Availability of EmNOC protocols and resuscitation drills

Maternal Health Guidelines prescribe the presence of EmNOC protocols across all facilities, and these have been developed at both the national and provincial levels [33]. Overall, health facilities performed poorly on the availability of these protocols as the mean score for the district was 41.0% ± 18.0%, with a range of 0.0% - 70.0%. The most readily available protocols in facilities were those of eclampsia (93.3%) and postpartum haemorrhage (86.7%). Only one facility had protocols on the management of retained placenta and shoulder dystocia (Fig 1). CHCs performed slightly better than hospitals, although some hospitals argued that the absence of protocols was not critical as they had obstetricians to provide guidance when needed. 73.3% of facilities had performed at least one neonatal resuscitation drill in the previous year, but only 6.7% had undertaken an adult resuscitation drill. Most facilities (93.3%) held regular obstetric and perinatal mortality and morbidity meetings.

## Discussion

All facilities in the study district performed some EmNOC life-saving functions. However, only hospitals performed all the required signal functions to be classified as CEmNOC facilities. The same hospitals provided all basic EmNOC services as well. None of the CHCs performed all required EmNOC signal functions to earn classification as basic EmNOC facilities. The overall target number of EmNOC facilities was 25, yet only 7 were present giving a shortfall of at least 18 facilities for the current district population. When births were used in the denominator rather than the general population, 16 facilities were required and the deficit was 9. The shortfall was more pronounced for basic EmNOC services. In addition, all facilities did not achieve optimal scores on nearly all checklists. Neonate resuscitation drills were performed by most facilities (73.3%) while only one facility performed adult resuscitation drills indicating absence of an important safeguard to quality of EmNOC care. While midwives were available in all facilities, advanced midwives were not always available 24 hours a day, 7 days a week. Doctors were available in CHCs only some days of the week. The mean workload for midwives (deliveries per midwife) in hospitals (36.4 ± 14.3) was disproportionately higher than that of CHCs (7.9 ± 3.2) and this difference was significant (p<0.01).

A limitation of this study was that only public health facilities were included. These results therefore show availability of EmNOC for public patients in the district. Adjustment of the population for those without medical insurance to estimate the population that relies on public facilities for care is common practice for planning purposes in South Africa [35, 36]. Although uninsured woman may fund private medical services out of pocket at times, this is less likely for expensive delivery services particularly when they are exempt from user fees in public facilities [37]. This study focused only on the first UN process indicator (Table 1). Evaluation of all process indicators would provide a more comprehensive assessment of EmNOC services in the district. However, inconsistent recording of obstetric emergencies in facility maternity registers at present prohibit the valid estimation of the important ‘met need’ or direct obstetric case fatality rates indicators for the district [38]. This prospective data collection in all facilities which was beyond the scope of this evaluation. Nevertheless, measuring the number of EmNOC facilities available remains important information for planning by policy makers and is part of EmNOC monitoring [10, 11, 19–21]. The Caesarean section rate is a routine district indicator [35] and there are national systems for the identification of maternal and neonatal mortality[24, 39].

As confirmed in this study, different denominators proposed to estimate the required density of EmNOC can produce significantly different results. The UN agencies have been criticised for inconsistently using both population and birth denominators interchangeably [10, 11, 40]. Populations with high fertility logically require more EmNOC facilities than similar sized populations with low fertility, rendering the births denominator more accurate for calculating the number of facilities required [10, 12, 41, 42]. In this study as the population denominator showed a much higher shortfall in EmNOC facilities than the births denominator for the same district. The births denominator seems more relevant for South Africa, as the country has one of the lowest fertility rates in sub-Sahara Africa at 2.41 births per woman [43, 44].

The availability of EmNOC in the literature is currently only measured by the number of EmNOC facilities [10, 15, 19, 38, 45, 46]. The capacity of these facilities in terms of size or numbers of delivery beds or numbers of women for whom EmNOC care can be provided are important considerations. But valid metrics for evaluating this capacity remain largely unexplored in the literature which is a critical gap.

Consensus on the package of life-saving functions that constitute basic and comprehensive EmNOC is important for planning purposes and as a basis for comparison [21, 41, 47–50]. While useful, the organisation of EmNOC services implied by the UN classification may not match low and middle income country (LMIC) settings. Studies show that many lower level facilities in LMICs are unable to provide all the signal functions required for basic EmNOC [17, 51]. Scholars have observed that signal functions that require more resource intensive (e.g. skilled providers, specialised equipment, infrastructure e.g. operating theatre) are performed less in many LMICs [51, 52]. The BEmNOC signal functions may be allocated in different configurations across existing facilities depending on the distribution of required resources [17, 21, 40, 51].

In this study, district management policy did not support three of the BEmNOC functions to be provided at the CHC level, and prescribed referral to hospitals where required resources (specialised skills and equipment) were available, thereby centralising EmNOC. Performance of EmNOC services such as assisted vaginal delivery at the primary level without an operating theatre and neonatal resuscitation facilities was considered unsafe. This was despite national policy guidelines stipulating that all the BEmNOC signal functions should be available in CHCs [33]. The disparity between the policy prescriptions of central policymakers in national maternal health guidelines and the services local providers believe are feasible, has been observed in other studies [32, 53]. To avoid such discrepancies, it has been recommended that policymakers should pilot policies before their adoption and scale-up [54]. It is also important to plan for the resources required to implement the policy [55].

On the other hand, centralising the provision of certain BEmNOC signal functions to secondary and tertiary levels of care may compromise coverage and access to essential EmNOC services [56, 57]. The centralisation-decentralisation tension typically ensues as it is often not feasible to decentralise all services to lower levels of care, particularly where resource scarcity prevails [58]. Furthermore, centralisation of all EmNOC to comprehensive facilities (hospitals) may not be the most efficient use of resources as more women would have to make use of relatively expensive hospital care for basic health needs that could have been provided more cheaply at lower levels of care. Moreover, for EmNOC services to be truly available to women when needed, coverage, and access of good quality EmNOC that is safe cannot be ignored [11, 19, 20, 32, 59]. It is necessary, therefore, to strike a balance between restricting performance of some signal functions to facility levels that guarantee patient safety and quality of care without compromising service coverage and access.

Additional signal functions (e.g. intravenous fluid administration for treating hypovolemic shock) have been proposed particularly for LMIC settings to provide more comprehensive measurement of the functionality of EmNOC facilities, [11, 16]. In this study these additional indicators proved important as some facilities did not attain full scores even for basic fluids that should have been readily available in any general health facility. General measures of facility functioning (e.g. opening hours, electricity, skilled providers) also gave important information on the ability of facilities to perform EmNOC and should be part of availability of EmNOC availability assessments as suggested by others [11, 16]. The UN EmNOC guidelines do not provide staffing norms for the distribution of skilled providers required to achieve availability of the signal functions in facilities across populations [10, 38, 60]. This study, for example, showed inequity in skilled staff distribution in relation to the workload across the district. Pattinson found similar discrepancies in the distribution of midwives in districts due to overstaffing in some facilities and understaffing in others [32].

In addition, there are no clear recommendations for how the quality of EmNOC services could be incorporated into the measurement of EmNOC availability. We included the availability of EmNOC protocols and the performance of EmNOC resuscitation drills in our facility evaluations. These could constitute more quality-orientated EmNOC signal functions as they have been shown to be useful in addressing avoidable maternal mortality [20, 24].

## Conclusions

The UN EmNOC signal functions were found to be useful for measuring the availability of EmNOC in the district to inform planning by provincial and district policymakers. The analysis revealed a deficit of EmNOC facilities, particularly basic EmNOC facilities. CHCs did perform some basic signal functions, but not all required to earn EmNOC facility classification. District policy restricted some signal functions to higher level facilities, and this led to centralisation of EmNOC. To improve the sensitivity of the availability of EmNOC indicator, studies measuring EmNOC availability need to consider facility opening hours, capacity and skilled staffing in addition to the demonstrated performance of signal functions. Furthermore, the use of births rather than population as the denominator in estimating the required density of EmOC facilities seems more applicable, particularly in countries with low fertility such as South Africa. We also advocate for the development and inclusion of indicators that better reflect the actual quality of available EmNOC care, as well as the capacity of EmNOC facilities in future EmNOC availability assessments.
